# Clinical characteristics and exploratory factors associated with in-hospital mortality in pediatric severe viral encephalitis

**DOI:** 10.3389/fped.2026.1957226

**Published:** 2026-09-30

**Authors:** Ping He, Fangqi Hu, Fei Wang

**Affiliations:** Department of Pediatrics, Anqing Municipal Hospital, Anqing, Anhui, China

**Keywords:** children, clinical characteristics, exploratory factors, in-hospital mortality, severe viral encephalitis

## Abstract

**Objective:**

The aim of this study was to describe the clinical characteristics of severe viral encephalitis (SVE) in children and to identify exploratory factors associated with in-hospital mortality, thereby providing preliminary evidence to facilitate early risk stratification and reduce SVE-related disability and mortality.

**Methods:**

A retrospective study was conducted in 75 children diagnosed with viral encephalitis at the Department of Pediatrics, Anqing Municipal Hospital, from January 2023 to December 2025, including 50 mild cases and 25 SVE cases. Demographic data, clinical manifestations, and laboratory parameters (peripheral white blood cell count (WBC), serum sodium, cerebrospinal fluid (CSF) leukocyte and protein levels, serum neuron-specific enolase (NSE), electroencephalography (EEG), neuroimaging findings, and Glasgow Coma Scale (GCS) scores) were collected and compared between groups. Pathogen detection was conducted through multiplex PCR and/or serum viral IgM antibody testing. In the mild encephalitis group, 14 out of 50 cases (28%) were found to be infected with specific viruses, with enteroviruses (EVs) being the predominant type, accounting for 7 cases, followed by herpes zoster virus (VZV) and human herpesvirus type 6 (HHV-6), each with 2 cases. In the SVE group, 9 out of 25 cases (36%) were found to have pathogens, with influenza A virus (4 cases) and herpes simplex virus (2 cases) being the main ones. Univariate analyses and Firth-penalized logistic regression were used to explore factors associated with in-hospital mortality (primary outcome) within the SVE subgroup.

**Results:**

Among the 25 children with SVE (15 males, mean age 7.74 ± 2.2 years), fever (76%), signs of elevated intracranial pressure (68%), and seizures (56%) were common; four patients died (16%). Compared with children with mild encephalitis, the SVE group exhibited significantly longer hospital stays, higher seizure rates, lower GCS scores, higher proportions of abnormal EEG and neuroimaging abnormalities, and elevated serum NSE (all *P* < 0.05). However, there were no significant differences in CSF leukocytes, CSF protein, blood leukocytes, or serum sodium. In univariate Firth-corrected logistic regression, neuroimaging abnormalities (*P* = 0.007), seizures (*P* = 0.011), EEG abnormalities (*P* = 0.018), lower GCS score (per 1-point decrease, *P* < 0.001), and higher serum NSE (per 10 ng/mL increment, *P* = 0.018) were significantly associated with in-hospital mortality.

**Conclusion:**

Pediatric SVE presents with nonspecific early symptoms and substantial mortality. Seizures, EEG and neuroimaging abnormalities, low GCS score, and elevated serum NSE are exploratory factors associated with in-hospital mortality. However, given the small sample size and single-center design, these findings are hypothesis-generating and require validation in larger multi-center prospective cohorts. Early recognition of these factors may aid in clinical risk assessment.

## 1 Introduction

Viral encephalitis is one of the most common central nervous system (CNS) infectious diseases in children. The disease progresses rapidly with diverse manifestations; severe cases often have poor outcomes (1). The incidence of viral encephalitis in children is significantly higher than in adults. Based on data from Henan Province during 2012–2023, approximately 4.5% of pediatric viral encephalitis cases progress to severe viral encephalitis (SVE), which was defined in that study as cases requiring pediatric intensive care unit (PICU) admission or presenting with altered consciousness or status epilepticus (2). SVE is characterized by acute onset, rapid progression, and high mortality (up to 8.4%) (3); some patients may evolve into acute necrotizing encephalopathy (4). Among survivors, about 20%–50% suffer from severe neurological sequelae (5), such as epilepsy, cognitive impairment, motor dysfunction, and behavioral abnormalities, which impose a long-term and heavy economic and psychological burden on families and society. Similar findings were reported by Gupta et al. in a retrospective study of pediatric acute encephalitis syndrome from eastern Uttar Pradesh, India, where a substantial proportion of survivors experienced long-term disability, including motor impairment and cognitive deficits (6). Qun Li *et al*. found that the incidence of sequelae, the number of patients receiving ICU treatment, and the number of deaths in the severe VE group were all significantly higher than those in the mild VE group (7). At present, the diagnosis of pediatric SVE mainly relies on clinical manifestations, cerebrospinal fluid (CSF) examination, and neuroimaging (8), but these methods have certain limitations. Therefore, early identification of SVE and understanding of exploratory factors associated with in-hospital mortality are crucial for timely and aggressive treatment. In the present study, we analyzed the clinical characteristics and ancillary examination findings of children with SVE to identify potential exploratory factors associated with in-hospital mortality using univariate analyses (with Firth-penalized-likelihood estimation to address complete separation), with the aim of improving clinicians’ understanding of pediatric SVE and reducing SVE-related mortality and disability.

## 2 Subjects and methods

### 2.1 Study subjects

All cases were derived from pediatric inpatients diagnosed with viral encephalitis at the Department of Pediatrics, Anqing Municipal Hospital from January 2023 to December 2025. All enrolled children fulfilled comprehensive diagnostic criteria for viral encephalitis as follows: (1) Acute onset of neurological symptoms and signs; (2) CSF examination showing an elevated WBC count (> 10 × 10⁶/L) with a predominance of lymphocytes, and/or a mild increase in protein; (3) Electroencephalogram and/or cranial imaging showing changes consistent with viral encephalitis; (4) Other central nervous system diseases such as purulent meningitis, tuberculous meningitis, cryptococcal meningitis, and autoimmune encephalitis were excluded. Alternative central nervous system infections were excluded according to the following criteria: 1) Bacterial purulent meningitis: excluded based on CSF profiles (predominance of polymorphonuclear leukocytes, low glucose, elevated protein), negative bacterial culture, and absence of clinical response to empirical antibiotic therapy. 2) Tuberculous meningitis: Excluded by CSF findings (lymphocytic pleocytosis, markedly elevated protein, decreased glucose), negative *Mycobacterium tuberculosis* PCR, and clinical response to antiviral therapy rather than anti-tuberculous therapy. 3) Cryptococcal meningitis: Excluded by negative India ink stain and negative cryptococcal antigen test in CSF. 4) Autoimmune encephalitis: Excluded by negative testing for anti-neuronal antibodies (anti-NMDA, anti-LGI1, anti-CASPR2, anti-GAD) in both serum and CSF.

In terms of pathogen detection, all children provided blood and CSF samples that were tested using multiplex PCR method or serum virus IgM antibody detection to detect viruses: herpes simplex virus 1/2 type, varicella-zoster virus, cytomegalovirus, Epstein–Barr virus (EBV), human herpesvirus 6 type; at the same time, throat swabs were collected for respiratory virus PCR detection (including influenza virus A/B, human parainfluenza virus, SARS-CoV-2). Pathogen detection in CSF and blood was performed using the Multiplex PCR Kit (Anhui Ranchi Biotechnology Co., Ltd., China), which detects HSV-1/2, VZV, and HHV-6. Respiratory viruses were detected from throat swabs using the Respiratory Virus PCR Kit (Guojun Group Anqing Co., Ltd., China), which includes influenza A/B, human parainfluenza virus, adenovirus, respiratory syncytial virus, and SARS-CoV-2. Serum virus-specific IgM antibodies were measured using commercial ELISA kits (Hefei Anmeson Medical Devices Co., Ltd., China). Notably, pathogen detection rates are influenced by multiple factors, including sampling timing, viral load, and primer design. A negative pathogen assay result does not exclude a clinical diagnosis of viral encephalitis (9). In cases where pathogen testing was negative, a clinical diagnosis of viral encephalitis was accepted based on the comprehensive criteria described above (acute neurological symptoms, CSF pleocytosis with lymphocytic predominance, compatible EEG/neuroimaging findings, and exclusion of other CNS diseases).

The patients were divided into two groups: a mild viral encephalitis group (*n* = 50) and an SVE group (*n* = 25). All patients met the diagnostic criteria for pediatric severe viral encephalitis as defined by a practical review on diagnostic approach and treatment. The diagnostic criteria for mild viral encephalitis were based on reference (10): All of the following clinical features must be met: 1) Consciousness state: Glasgow Coma Scale (GCS) ≥ 13 points (drowsy or agitated, but able to be awakened, and able to follow commands); 2) Seizure episodes: No seizures, or only single, brief convulsive episodes; 3) Focal signs: No obvious limb paralysis, cranial nerve palsy, or ataxia (or extremely mild and short-term recovery); 4) Intracranial hypertension: No severe headache, projectile vomiting, papilledema, and normal fundus examination; 5) Vital signs: Stable respiration, heart rate, and blood pressure, no need for ventilator support; 6) Daily abilities: Can eat independently, with basic life skills retained. The diagnostic criteria for SVE were adopted without modification from reference (7) and included at least one of the following conditions: (1) Frequent convulsions or status epilepticus; (2) Consciousness disturbance with a Glasgow Coma Scale (GCS) score < 8; (3) Limb paralysis or neurobehavioral abnormalities; (4) Brainstem symptoms; (5) Severe or persistent intracranial hypertension; (6) Multiple organ impairment progressing to failure. Because seizures and low GCS are components of the SVE definition, their associations with mortality within the SVE subgroup should be interpreted as severity markers rather than independent predictors. This study was approved by the ethics committee of Anqing Municipal Hospital (2024-94).

Grouping based on GCS and seizures introduces definition-dependent differences between mild and severe cases; thus, comparisons serve only to describe clinical profiles, not to validate diagnostic values. In the prognostic regression analysis, whose primary outcome is in-hospital mortality within the SVE group, these variables are included as potential exploratory factors. However, because seizures and low GCS are components of the SVE definition, their association with mortality should be interpreted with caution, as discussed in the discussion section. Discharge-level functional prognosis was evaluated using the Glasgow Outcome Scale (GOS): 1 = death, 2 = persistent vegetative state, 3 = severe disability, 4 = moderate disability, 5 = good recovery. Poor outcome was defined as GOS 1–3, whereas favorable outcome corresponded to GOS 4–5. GOS was collected for descriptive functional-status summarization and was not adopted as the primary endpoint in prognostic regression models.

### 2.2 Data collection

Early indicators were collected from children with viral encephalitis within 24–48 h after admission, as described below. All variables included in the prognostic regression analysis were measured at admission or within 24 h of admission, prior to knowledge of the clinical outcome.

#### 2.2.1 Clinical symptoms and signs (assessed at admission or within 24 h after admission)

1) Level of consciousness: The adapted Pediatric Glasgow Coma Scale (GCS) score (11)—a core indicator of neurological function. A GCS score ≤ 12 or a rapid decline in GCS suggests severe disease. All assessors were pediatric critical-care specialists of attending-physician rank or higher. Pediatric GCS scores were independently assigned and cross-validated by two assessors (physicians and nurses). All assessors had passed the standardized training and assessment within the department. The results of the two assessments were consistent (with a total score difference of ≤ 1 point), and the results are reliable. 2) Seizure manifestations: Presence of convulsive status epilepticus, frequent seizures (≥ 3 episodes within 24 h); or focal seizures suggesting damage to a specific brain region. 3) Signs of increased intracranial pressure: Vomiting (especially projectile); bulging anterior fontanel (in infants). 4) Motor dysfunction: Hemiplegia or significant abnormalities in muscle tone (hypertonia or hypotonia). 5) Systemic manifestations: Hyperthermia (e.g., body temperature > 39 °C or difficult-to-control fever); hypothermia (may indicate severe sepsis or brainstem damage).

#### 2.2.2 Routine laboratory indicators (initial test results)

1) Peripheral blood test: White blood cell (WBC) count—a significantly elevated or decreased count may indicate severe disease. 2) Serum sodium: hyponatremia, especially < 130 mmol/L, strongly suggests possible syndrome of inappropriate antidiuretic hormone secretion (SIADH), which is a marker of increased intracranial pressure and disease worsening. 3) Cerebrospinal fluid (CSF) analysis: WBC count—a high count does not necessarily indicate more severe disease; Protein content—a significant elevation (> 1 g/L) indicates severe disruption of the blood-brain barrier.

#### 2.2.3 Biomarkers of neurological injury

Serum neuron-specific enolase (NSE), which reflects neuronal injury. Within one hour of the patient's admission, blood samples were collected for testing (Shanghai Fudai Testing Technology Group Co. Ltd.). The double-antibody sandwich chemiluminescence immunoassay method was used. The laboratory reference range for serum NSE in healthy children in our institution is 0–16.3 ng/mL. A threshold of > 25.0 ng/mL was predefined by the manufacturer and supported by previous literature as indicating clinically significant neuronal injury, and was used to define “elevated” NSE in the descriptive analyses. For the regression analysis, serum NSE was modeled as a continuous variable per 10 ng/mL increment.

#### 2.2.4 Early imaging and neurophysiological examinations (performed within 24 h)

1) Neuroimaging: Neuroimaging was performed in all 75 enrolled patients. Cranial MRI was performed in 22 SVE patients and 45 mild VE patients; 3 SVE patients underwent CT because of their clinical condition. MRI abnormalities were defined as single or multiple abnormal signal intensities on T1- and T2-weighted sequences, with or without contrast enhancement. CT abnormalities were defined as focal or diffuse hypodensities or cerebral edema. To maintain analytical consistency in the analysis, “neuroimaging abnormalities” included findings from either MRI or CT. 2) Electroencephalography (EEG): A 30-minute monitoring session was performed. Abnormal EEG findings included widespread theta or delta slow waves (< 4 Hz), persistent asymmetry in wave amplitudes on both sides (one side decreased > 50% or increased > 100%), loss of reactivity, sharp waves, spike waves: sharp-slow or spike-slow complex waves were considered abnormal.

### 2.3 Statistical analysis

Statistical analyses were performed using SPSS version 20.0. Normality of continuous variables was assessed using the Shapiro–Wilk test. Continuous variables with a normal distribution were expressed as mean ± standard deviation (SD), and inter-group comparisons were performed using the independent-samples t-test. Non-normally distributed continuous variables were expressed as median with interquartile range [IQR], and inter-group comparisons were performed using the Mann–Whitney U test. Categorical variables were expressed as frequency (n) and percentage (%), and comparisons were performed using the chi-square test or Fisher's exact test. Univariate analysis was performed to identify exploratory factors associated with in-hospital mortality in children with SVE. Univariate logistic regression with Firth's penalized-likelihood estimation was implemented for bias correction. All statistical tests were two-tailed, and *P* < 0.05 was regarded as the threshold for statistical significance.

The statistical analysis was conducted in two distinct parts. First, descriptive comparisons of demographic, clinical, and laboratory characteristics were performed between the mild encephalitis and SVE groups to illustrate the clinical profile of SVE (Tables 1, 2). Second, a prognostic analysis was performed exclusively within the SVE subgroup (*n* = 25), using univariate Firth-penalized logistic regression to explore factors associated with the primary outcome of in-hospital mortality (Table 3). The variables included in the regression models were pre-specified as age, sex, GCS score, presence of seizures, EEG abnormalities, neuroimaging abnormalities, and serum NSE levels.

**Table 1:** Demographic and Clinical Characteristics of Children with Mild and Severe Viral Encephalitis.

Variable	Mild viral encephalitis (*n* = 50)	Severe viral encephalitis (*n* = 25)	*P*-value
Demographic			
Age (years), mean ± SD	5.80 ± 2.20	7.74 ± 2.20	<0.01
Male, *n* (%)	27 (54.00)	15 (60.00)	0.62
Season (Winter-Spring), *n* (%)	21 (42.00)	17 (68.00)	0.03
Prior Health Status, *n* (%)	47 (94.00)	24 (96.00)	0.72
Clinical Presentation			
Length of hospitalization (d), mean ± SD	9.68 ± 3.09	13.28 ± 4.82	<0.01
Length of fever (d), mean ± SD	2.24 ± 2.51	3.12 ± 2.65	0.16
GCS score, mean ± SD	14.22 ± 0.91	9.96 ± 3.07	<0.01
Seizures, *n* (%)	14 (28.00)	14 (56.00)	0.02
Fever, *n* (%)	40 (80.00)	19 (76.00)	0.69

**Table 2:** Comparison of laboratory indicators between children with SVE and those with mild viral encephalitis.

Variable	Mild viral encephalitis (*n* = 50)	Severe viral encephalitis (*n* = 25)	*P*-value
EEG abnormalities, *n* (%)	20 (40.0)	21 (84.0)	<0.01
Neuroimaging abnormalities, *n* (%)	8 (16.0)	11 (44.0)	<0.01
CSF leukocyte count (×10⁶/L), median [IQR]	26 [8–87]	68 [12–134]	0.16
CSF protein (mg/L), median [IQR]	247 [146–580]	982 [354–1,326]	0.21
Peripheral blood WBC (×10⁹/L), mean ± SD	12.56 ± 17.70	9.39 ± 4.13	0.38
Serum sodium (mmol/L), mean ± SD	137.41 ± 5.56	136.62 ± 4.13	0.53
Serum NSE (ng/mL), median [IQR]	37 [16–49]	48 [30–84]	<0.01

Data are presented as mean ± SD, median [IQR], or *n* (%). *P*-values were calculated using the independent-samples t-test for normally distributed continuous variables, the Mann–Whitney U test for non-normally distributed continuous variables, and the chi-square or Fisher's exact test for categorical variables. This table presents a descriptive comparison between mild and severe cases and was not interpreted as a prognostic analysis.

**Table 3:** Univariate comparisons and Firth-penalized logistic regression for exploratory factors associated with in-hospital mortality in children with SVE.

Variable	Survival group (*n* = 21)	Death group (*n* = 4)	Univariate *P*-value	OR (95% CI)	Regression *P*-value
Seizures	10 (47.6)	4 (100.0)	0.06	207.00 (9.87–4321.00)	0.01
EEG abnormalities	17 (81.0)	4 (100.0)	0.35	81.00 (3.65–1793.00)	0.02
Neuroimaging abnormalities	7 (33.3)	4 (100.0)	0.02	261.00 (12.34–5517.00)	<0.01
GCS score	10.76 ± 2.45	5.75 ± 1.50	<0.01	2.16 (1.39–3.36)^#^	<0.01
Serum NSE (ng/mL)	42.0 [28.0–68.0]	96.0 [72.0–128.0]	<0.01	1.58 (1.07–2.32)*	0.02
Duration of fever (d)	2.95 ± 2.54	4.00 ± 3.56	0.53	—	—
CSF leukocyte count (×10⁶/L)	64.0 [12.0–112.0]	128.0 [42.0–178.0]	0.18	—	—
CSF protein (mg/L)	864.0 [312.0–1,180.0]	1 326.0 [846.0–1,842.0]	0.10	—	—
Peripheral blood WBC (×10⁹/L)	9.62 ± 4.39	8.23 ± 2.63	0.55	—	—
Serum sodium (mmol/L)	137.05 ± 4.21	134.25 ± 2.63	0.21	—	—

Data are presented as mean ± SD, median [IQR], or *n* (%). *P*-values were calculated using the independent-samples t-test for normally distributed continuous variables, the Mann–Whitney U test for non-normally distributed continuous variables, and the chi-square or Fisher's exact test for categorical variables. ^#^OR per 1-point decrease in GCS score (i.e., lower GCS = higher mortality risk). *OR per 10 ng/mL increment in serum NSE. “—” indicates that the variable was not included in the logistic regression. The primary outcome for this analysis was in-hospital mortality (death versus survival).

## 3 Results

### 3.1 General characteristics

Among the 50 children with mild viral encephalitis, there were 27 males and 23 females, with a mean age of 5.8 ± 2.2 years. Mild cases occurred year-round; 21 (42%) presented in winter-spring (December-May) and 29 (58%) in summer-autumn (June-November). Among the 25 SVE patients, there were 15 males and 10 females, with a mean age of 7.74 ± 2.2 years. SVE also occurred throughout the year, with 17 cases (68%) in winter-spring and 8 cases (32%) in summer-autumn. Acute onset (≤ 7 days) was observed in 23 cases (92%), and subacute onset (> 7 days) in 2 cases (8%). Compared with the mild viral encephalitis group, the SVE group had significantly longer hospital stays, higher seizure rates and lower GCS scores (all *P* < 0.05), while the incidence of fever was not significantly different between the two groups (*P* > 0.05). See Table 1.

All 75 children (50 mild, 25 SVE) underwent blood and CSF sampling for pathogen detection using multiplex PCR and/or serum virus IgM antibody tests. Additionally, throat swabs for respiratory virus PCR were collected for all patients. In the mild encephalitis group, 14 out of 50 cases (28%) were found to be infected with specific viruses. Among them, 7 cases were infected with enteroviruses (EVs), followed by VZV and human herpesvirus type 6 (HHV-6), each with 2 cases; 1 case of influenza A virus; 1 case of parainfluenza virus; and 1 case of EBV. Among the 25 SVE patients, 4 were infected with influenza A, 2 with HSV, 1 with SARS-CoV-2, 1 with human parainfluenza, 1 with adenovirus, and the remaining cases had no specific virus identified (clinical diagnosis was accepted as per the criteria described in Methods).

### 3.2 Clinical symptoms and signs (SVE)

Fever was the most frequent clinical symptom, observed in 19 out of 25 patients (76%), followed by signs of elevated intracranial pressure (e.g., headache and vomiting) in 17 patients (68%). Neurological dysfunction, including limb shaking, unsteady gait, hemiplegia, or slurred speech, was the initial manifestation in 8 patients (32%). Respiratory symptoms occurred during the disease course in 15 patients (60%). Psychobehavioral abnormalities, mainly excitatory symptoms such as irritability and delirium, were present in 6 patients (24%). Altered consciousness was observed in 11 patients (44%), and cognitive impairment was noted in a small proportion of cases (8%). Seizures occurred in 14 patients, predominantly as generalized seizures.

### 3.3 Comparison of laboratory indicators between pediatric SVE and mild viral encephalitis

Compared with the mild viral encephalitis group, the SVE group had significantly longer hospital stays, higher rates of seizures, and lower GCS scores (all *P* < 0.05). Regarding laboratory indicators, the SVE group showed significantly higher rates of EEG abnormalities, neuroimaging abnormalities, and serum NSE levels than the mild encephalitis group (all *P* < 0.05). Among the 25 patients with SVE, 22 underwent cranial MRI and 3 underwent cranial CT because of their clinical condition. Eleven patients (44%) had abnormal neuroimaging findings: 10 on MRI and 1 on CT. MRI abnormalities presented as single or multiple patchy or band-like long T1 and long T2 signals, and some were accompanied by inflammatory demyelination. The MRI lesions involved the unilateral insular lobe in 4 cases, the brainstem in 2, the cerebellum in 1, the bilateral frontal lobes in 4, and extensive brain parenchyma in 2. EEG was abnormal in 21 cases: 11 showed paroxysmal diffuse high-amplitude slow waves, and 10 had focal epileptic discharges. However, no significant differences were observed in CSF leukocyte count, CSF protein, blood leukocyte count, or serum sodium (all *P* > 0.05). See Table 2.

### 3.4 Univariate comparisons and firth-penalized logistic regression for factors associated with in-hospital mortality in children with SVE

The primary outcome for this analysis was in-hospital mortality (death versus survival during hospitalization). Among continuous variables, the death group had significantly lower GCS scores (*P* = 0.001) and significantly higher serum NSE levels (*P* = 0.007) compared with the survival group. No significant differences were observed in length of fever, CSF leukocyte count, CSF protein, peripheral blood WBC, or serum sodium between the two groups (all *P* > 0.05).

Among the examined variables, neuroimaging abnormalities showed the strongest association with mortality (*P* = 0.007), followed by seizures (*P* = 0.011) and EEG abnormalities (*P* = 0.018). For continuous variables, each 1-point decrease in GCS score was associated with a significantly increased risk of death (*P* < 0.001), meaning that lower GCS scores (more severe neurological impairment) were strongly related to higher mortality. Similarly, each 10 ng/mL increment in serum NSE was associated with an increased in-hospital mortality risk (*P* = 0.018). The odds ratio for GCS is interpreted per 1-point decrease in the score (lower GCS → higher mortality risk). The odds ratio for NSE is interpreted per 10 ng/mL increment. No significant associations were found for length of fever, CSF leukocyte count, CSF protein, peripheral blood WBC count, or serum sodium (all *P* > 0.10). See Table 3.

### 3.5 Treatment and prognosis of children with severe viral encephalitis

Upon clinical suspicion of viral encephalitis, all patients received intravenous acyclovir (10–15 mg/kg per dose, administered every 8 h), usually within 2-4 h after admission and prior to definitive microbiological confirmation. Corticosteroid therapy (methylprednisolone 10 mg/kg/day for 3 days) and/or intravenous immunoglobulin (IVIG, 400 mg/kg/day for 3 days) were administered at the discretion of the treating physician based on clinical severity, as indicated by GCS score ≤ 12, refractory seizures, or progressive neurological deterioration, typically initiated within 24 h of SVE diagnosis.

In the SVE group, 21 of 25 children (84%) achieved recovery after treatment. All patients in both groups received intravenous acyclovir as standard antiviral therapy. Treatment allocation differed between survivors and non-survivors. Among survivors, 18/21 (85.7%) received corticosteroid (either alone or in combination), and 13/21 (61.9%) received combined corticosteroid + IVIG therapy. Among non-survivors, 2/4 (50%) received corticosteroid, and 1/4 (25%) received combination therapy. Thus, corticosteroid monotherapy (i.e., without IVIG) was used in 5/21 (23.8%) of survivors and 1/4 (25%) of non-survivors. These differences reflect clinical judgment and severity at presentation rather than a randomized treatment effect. There were 2 cases of epilepsy and 1 case of decreased lower limb muscle strength among survivors; there were 4 deaths. See Table 4.

**Table 4:** Treatment and prognosis of children with severe viral encephalitis.

Treatment category	Mild viral encephalitis (*n* = 50)	Severe viral encephalitis – Survival (*n* = 21)	Severe viral encephalitis – Death (*n* = 4)	Severe viral encephalitis – Total (*n* = 25)
Corticosteroid use (any)	5 (10.0%)	18 (85.7%)	2 (50.0%)	20 (80.0%)
IVIG use (any)	2 (4.0%)	13 (61.9%)	2 (50.0%)	15 (60.0%)
Corticosteroid + IVIG (combined)	2 (4.0%)	13 (61.9%)	1 (25.0%)	14 (56.0%)
Corticosteroid alone (calculated)	3 (6.0%)	5 (23.8%)	1 (25.0%)	6 (24.0%)
Antiviral therapy (acyclovir)	50 (100%)	21 (100%)	4 (100%)	25 (100%)

Data are presented as *n* (%). “Corticosteroid alone” was calculated as the difference between “Corticosteroid use (any)” and “Corticosteroid + IVIG (combined)”, representing patients who received corticosteroids without IVIG. Percentages are calculated based on the group total (survival group *n* = 21; death group *n* = 4; total SVE *n* = 25).

## 4 Discussion

Viral encephalitis arises from viral invasion of the intracranial compartment, triggering diffuse parenchymal brain inflammation. Early clinical manifestations are heterogeneous and may be limited to non-specific symptoms including fever, headache, fatigue, and myalgia (12). The incidence of viral encephalitis shows a seasonal pattern, with peaks from June to July and from December to January (13), which is similar to the pattern observed in this study. The winter-spring predominance in our cohort may reflect the circulation pattern of respiratory viruses such as influenza and adenovirus, which constituted the majority of identified pathogens in this study. Unlike the classic predominance of enteroviruses reported in other pediatric encephalitis cohorts (14), our findings suggest that respiratory viruses may play a more prominent role in severe encephalitis cases during the winter-spring season in our region. Furthermore, we found that the age of children with severe encephalitis in Anqing was slightly older than that of those with mild conditions. In the present cohort, the pathogen detection rate among patients with SVE reached 36% (9/25). Influenza-A virus was the most frequently identified pathogen (4/25, 16%), followed by herpes simplex virus (2/25, 8%). This distribution was different from the classic pattern reported in the literature. It is possible that during this period, the enterovirus may not be the predominant circulating strain in this region. The sensitivity of viral nucleic acid testing is influenced by various factors such as sampling time, primer design, and viral load, approximately 40% to 60% of clinical cases of viral encephalitis have no identifiable pathogen, as documented in the literature (14).

In recent years, the onset of viral encephalitis has shown a trend toward younger age groups, with increased incidence in neonates and infants (15). If not diagnosed and treated promptly, children may develop severe neurological complications (16); therefore, early and accurate diagnosis is crucial for improving prognosis. In most cases, mild viral encephalitis has a good prognosis after active treatment, whereas severe viral encephalitis progresses rapidly, is more likely to cause other CNS symptoms, leads to sequelae, and can even result in death (7). Clinically, brain failure is common in severe viral encephalitis and is an important cause of death in pediatric intensive care units. In the acute phase of pediatric SVE, various symptoms may occur, including agitation, drowsiness, coma, neurological dysfunction, convulsions, and psychobehavioral abnormalities. The most common clinical symptoms of SVE are headache, decreased consciousness, and convulsions, and in most cases the viral etiology remains unknown (3, 12), which is consistent with the clinical presentation of SVE patients in this study. The Glasgow Coma Scale (GCS) is a rapid indicator of the severity of brain injury and plays an important role in determining patient prognosis. In the present study, univariate analyses demonstrated that children with SVE who died exhibited significantly lower GCS scores and higher serum NSE concentrations compared with survivors. Neuroimaging abnormalities were also markedly more prevalent among non-survivors. These observations align with prior reports demonstrating that the magnitude of brain injury reflected by GCS, NSE, and neuroimaging is tightly associated with prognosis in viral encephalitis (7, 17). Pratamastuti D *et al*. found that the median serum NSE level in the acute encephalitis syndrome was significantly higher than that of the non-acute encephalitis syndrome group, these results indicate that serum NSE was a good indicator of neuronal brain injury (18). Similarly, in our study, serum NSE levels were higher in children with SVE than in those with mild viral encephalitis, and elevated serum NSE was identified as an exploratory factor associated with in-hospital mortality. Abnormal EEG and neuroimaging findings, as well as high NSE levels, reflect the extent of brain tissue injury and help assess patient prognosis.

In the present study, most children with SVE received combined corticosteroid and immunoglobulin therapy, and the overall recovery rate was relatively high. In this cohort, immunomodulatory therapy was more frequently used in patients with more severe clinical presentations. The observational nature precludes causal inference, but our data provide a real-world snapshot of current management practices for pediatric SVE. In recent years, researchers have increasingly focused on exploring prognosis-associated factors in patients with established SVE, attempting to construct prediction models using clinical symptoms, laboratory indicators, and other parameters (16, 19). Gao et al. found that convulsive status epilepticus, stress-induced hyperglycemia, and miR-125b are exploratory factors associated with in-hospital mortality in children with SVE (20). Song et al. reported that levels of soluble interleukin-2 receptor (SIL-2R), endothelial nitric oxide synthase (eNOS), and CD93 in CSF and serum are significantly elevated in children with viral encephalitis and are closely associated with disease progression and prognosis, suggesting their potential value as early warning biomarkers (21). However, current research in this field is still in a preliminary stage, and a unified early warning indicator system with standardized cutoff values has not yet been established, limiting its clinical application and generalization. The key challenge in the clinical management of SVE is the mismatch between the therapeutic time window and the assessment of disease severity. This study demonstrated that seizures, EEG abnormalities, neuroimaging abnormalities, low GCS score, and elevated serum NSE level were significantly associated with in-hospital mortality in children with SVE. However, due to the small sample size (particularly the small number of deaths, *n* = 4), we were unable to perform multivariable adjustment for potential confounders. Therefore, these associations should be regarded as exploratory and preliminary rather than confirmatory. The extremely wide confidence intervals for categorical variables further reflect the statistical instability inherent in small-sample analyses. Larger multicenter studies with sufficient sample sizes are urgently needed to establish independent exploratory factors and to develop reliable risk stratification tools for pediatric SVE.

Treatment modalities for SVE include antiviral drugs, osmotic agents to reduce intracranial pressure, corticosteroid and immunoglobulins, antiepileptic drugs, brain protection, and brain metabolism-enhancing agents. Studies have reported that early use of hemoperfusion combined with continuous veno-venous hemodiafiltration may improve the prognosis of pediatric SVE more than hemoperfusion alone (22). The timing of therapy initiation was within 24–48 h of diagnosis, and the indications were based on clinical judgment (rapid neurological deterioration, brain edema on imaging, or refractory seizures). In this cohort, the majority of SVE patients received corticosteroids and/or IVIG, and 84% survived to discharge. However, the observational nature of this study precludes causal inference regarding treatment efficacy. Treatment allocation was based on clinical judgment and disease severity, and differences in treatment between survivors and non-survivors likely reflect confounding by indication. Therefore, the observed association between immunotherapy and survival should not be interpreted as evidence of treatment benefit. Prospective randomized studies are needed to evaluate the efficacy of immunomodulatory therapies in pediatric SVE. The role of immunotherapy in pediatric SVE remains an area of ongoing investigation (23).

Several limitations of this study must be acknowledged. First and foremost, this is a single-center retrospective observational study with a limited sample size (*n* = 25 for the SVE subgroup) and, critically, only four mortality events. This low event number constrains the statistical power and numerical stability of our regression models. While we employed Firth-penalized logistic regression to mitigate the issue of complete separation, the resulting effect estimates, particularly the wide confidence intervals for categorical variables, should be interpreted with considerable caution. Second, the exploratory nature of our analysis precludes any definitive conclusions about exploratory factors associated with in-hospital mortality, and our findings should be considered hypothesis-generating rather than confirmatory. Third, incorporation-circularity bias represents a potential limitation: seizures and altered consciousness (low GCS) are embedded within the SVE case definition. Their strong observed association with mortality among the SVE subgroup is partly inherent to the case definition. This circularity does not invalidate their clinical utility as markers of severity, but it necessitates caution when interpreting them as independent exploratory factors associated with in-hospital mortality. Our findings regarding these variables should be viewed as supportive of their established role in disease severity stratification rather than as novel prognostic discoveries. Fourth, as a single-center study in Anqing, China, the generalizability of our findings to other populations with different viral epidemiology and clinical practices is limited. Future large-scale, multicenter prospective studies are urgently needed to validate these preliminary findings and establish robust risk stratification tools.

## 5 Conclusion

The burden of pediatric SVE remains substantial. Early clinical symptoms are non-specific; specific clinical markers and sensitive early-warning tools are lacking, and clinical prognosis is often unfavorable. Convulsive seizures, abnormal EEG findings, neuroimaging abnormalities, elevated serum NSE concentrations, and low Glasgow Coma Scale (GCS) scores represent potential factors associated with in-hospital mortality and merit further validation for mortality-risk stratification among children with SVE. Based on the above background, this study describes the clinical characteristics (including demographics, symptoms, signs, laboratory findings, EEG and neuroimaging features) of children with SVE in Anqing City of China, and compares the clinical and laboratory profiles between SVE and mild viral encephalitis. Finally, we identified potential factors associated with in-hospital mortality in children with SVE using univariate analysis. The ultimate goal is to provide preliminary regional data that may help clinicians recognize SVE early and guide risk stratification, while acknowledging the exploratory nature of the findings.

## Data Availability

The original contributions presented in the study are included in the article/Supplementary Material, further inquiries can be directed to the corresponding author.
